# Lorazepam-Induced Orthostatic Hypotension and Secondary Headache in a Low Blood Pressure Phenotype

**DOI:** 10.7759/cureus.97894

**Published:** 2025-11-26

**Authors:** Sungmin Song, Yoobin Kang, Tanveer Padder

**Affiliations:** 1 Medicine, Trinity School of Medicine, Warner Robins, USA; 2 Psychiatry, TIME Organization, Baltimore, USA

**Keywords:** anticholinergic mucosal dryness, baroreflex suppression, benzodiazepine adverse effect, low blood pressure phenotype, orthostatic intolerance

## Abstract

A 30-year-old man with a lifelong low but stable blood pressure (BP) (typically 90-95/50-55 mmHg) developed dose-related orthostatic symptoms and evening headaches after the initiation of nightly lorazepam. Classic orthostatic hypotension (OH) thresholds were not consistently met; however, standing repeatedly reproduced symptoms with concurrent heart rate (HR) increases, a pattern more consistent with orthostatic intolerance (OI) time-locked to benzodiazepine (BZD) exposure. Symptoms were reliably worse on 2 mg nights and attenuated on 1 mg or off-nights (dechallenge/re-challenge). Co-exposure to a tricyclic antidepressant (TCA) and a first-generation antihistamine (AH) increased anticholinergic burden (ACB), aligning with nocturnal nasal dryness and ocular surface complaints. Baseline evaluation (complete blood count (CBC), comprehensive metabolic panel (CMP), thyroid function, hemoglobin A1c (HbA1c), electrocardiogram (ECG)) showed no secondary causes. Urinalysis (UA) on high-symptom days demonstrated higher specific gravity with intermittent ketonuria and hyaline casts (per low-power field (LPF)), suggesting relative dehydration as a modulator. The case highlights BZD-related autonomic dampening in a constitutional low BP phenotype and supports practical safeguards: individualized dosing, orthostatic BP/HR monitoring with symptom logging, reduction of total anticholinergic load, hydration/salt strategies, and cognitive behavioral therapy for insomnia (CBT-I)-first insomnia care.

## Introduction

Benzodiazepines (BZDs) are frequently prescribed for insomnia and anxiety because of their gamma-aminobutyric acid (GABA)-mediated sedative effects, but chronic exposure can alter autonomic nervous system (ANS) control of blood pressure (BP) and predispose to orthostatic symptoms [1]. Orthostatic hypotension (OH) is classically defined as a sustained decrease of systolic BP ≥20 mmHg or diastolic BP ≥10 mmHg within three minutes of standing and may reflect impaired baroreflex buffering or drug-induced vascular dysregulation [2]. In contrast, orthostatic intolerance (OI) refers to reproducible orthostatic symptoms (e.g., lightheadedness, visual blurring, chest tightness) accompanied by postural increases in heart rate (HR) and modest BP changes that may not reach these OH thresholds. BZDs can blunt sympathetic outflow and baroreflex sensitivity, providing a mechanistic basis for such postural BP and HR instability [3]. Observational data further show greater immediate postural BP drops and higher fall risk among BZD users, supporting a clinically significant impact on orthostatic responses [4].

Tricyclic antidepressants (TCAs) and first-generation antihistamines (AHs) add a second pharmacologic hit by increasing systemic anticholinergic burden (ACB), reducing exocrine secretions, and producing nasal and ocular dryness [5]. Chronic anticholinergic exposure reduces tear-film secretion and has been associated with ocular surface fragility and delayed corneal epithelial repair, an important consideration after corneal refractive surgery [6]. In this report, we describe a young man with constitutional low BP who developed dose-related OI and evening headaches after lorazepam initiation, later complicated by anticholinergic-associated mucosal and ocular symptoms under sedative polypharmacy. This case illustrates how BZDs and cumulative ACB can precipitate clinically significant orthostatic symptoms in low BP phenotypes and highlights practical safeguards for sedative prescribing in such patients.

## Case presentation

The patient is a 30-year-old man and a non-smoker, with no history of alcohol use. He has a lifelong history of low but stable BP (typically 90-95/50-55 mmHg), and his mother also reports lifelong constitutional hypotension, suggesting a possible inherited autonomic tendency, with an additional family history of type 2 diabetes. His insomnia began eight years ago during his frontline military service, where sustained psychological stress likely contributed. Non-BZD approaches, including sleep hygiene, melatonin, AHs, and selective serotonin reuptake inhibitors (SSRIs), were ineffective, prompting the initiation of nightly lorazepam 1 mg in March 2019. This provided partial symptomatic relief. However, after starting lorazepam, he began experiencing orthostatic-type symptoms such as transient blurred vision, chest tightness, and occasional lightheadedness upon standing, though syncope never occurred; these symptoms were later observed to be dose-related, worsening on 2 mg nights and attenuating on 1 mg or off-nights.

Routine home BP monitoring over several weeks revealed a consistent baseline BP of 90-95/50-55 mmHg, which is consistent with his hypotensive phenotype. Despite normal laboratory findings (complete blood count (CBC), comprehensive metabolic panel (CMP), thyroid function, hemoglobin A1c (HbA1c), electrocardiogram (ECG), etc.), the persistence of his symptoms suggested a medication-related mechanism.

**Figure 1:**
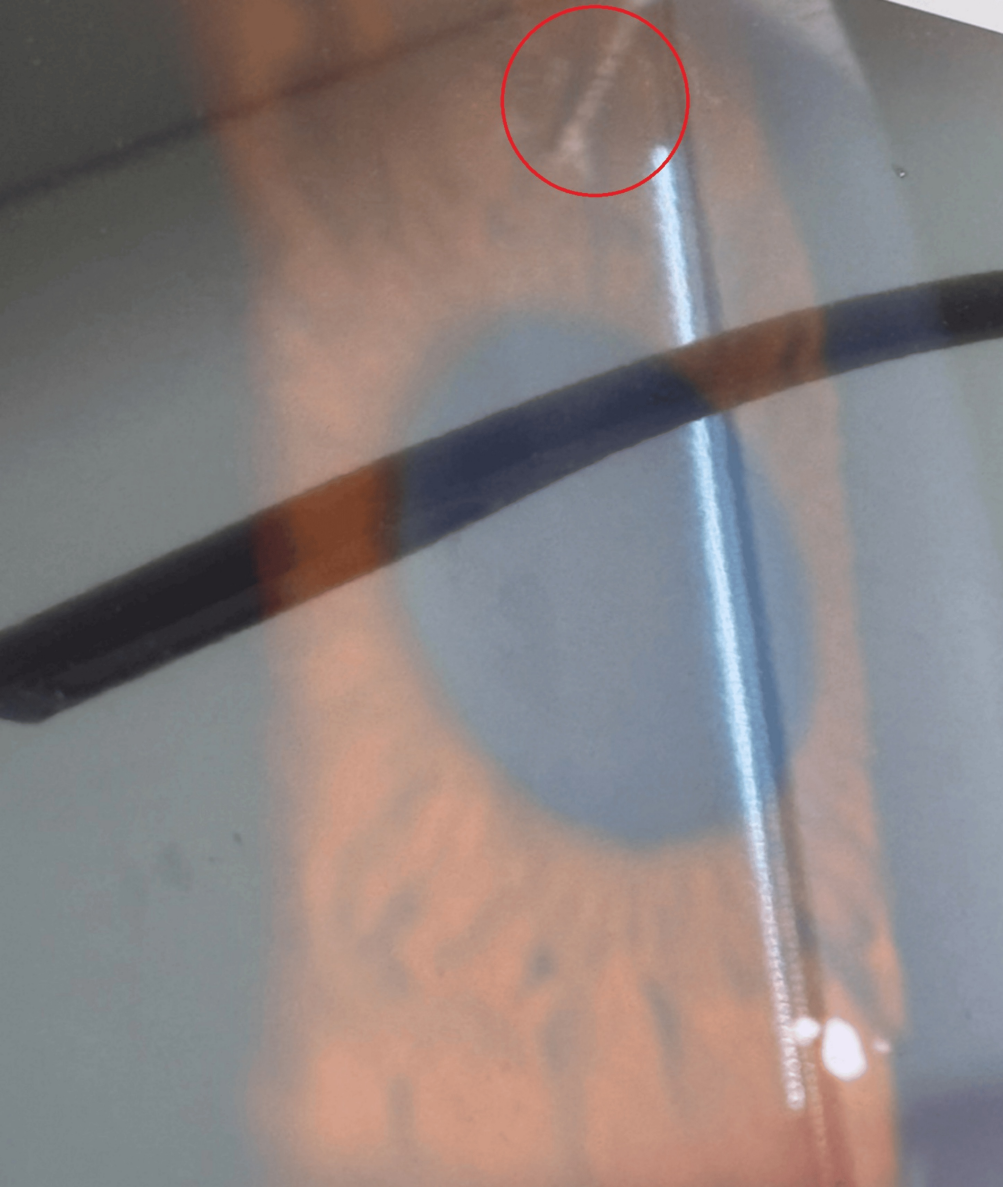
Initial inferior paracentral corneal epithelial defect (slit-lamp photograph), July 2024

In July 2024, the patient underwent laser-assisted in situ keratomileusis (LASIK). He had no prior history of clinically significant dry eye or recurrent corneal erosions, but following this procedure, he developed recurrent corneal epithelial erosions documented on slit-lamp examination (Figure 1). At that time, he was taking nightly lorazepam 2 mg as well as imipramine (tricyclic antidepressant) and diphenhydramine (first-generation AH) for breakthrough insomnia.

**Figure 2:**
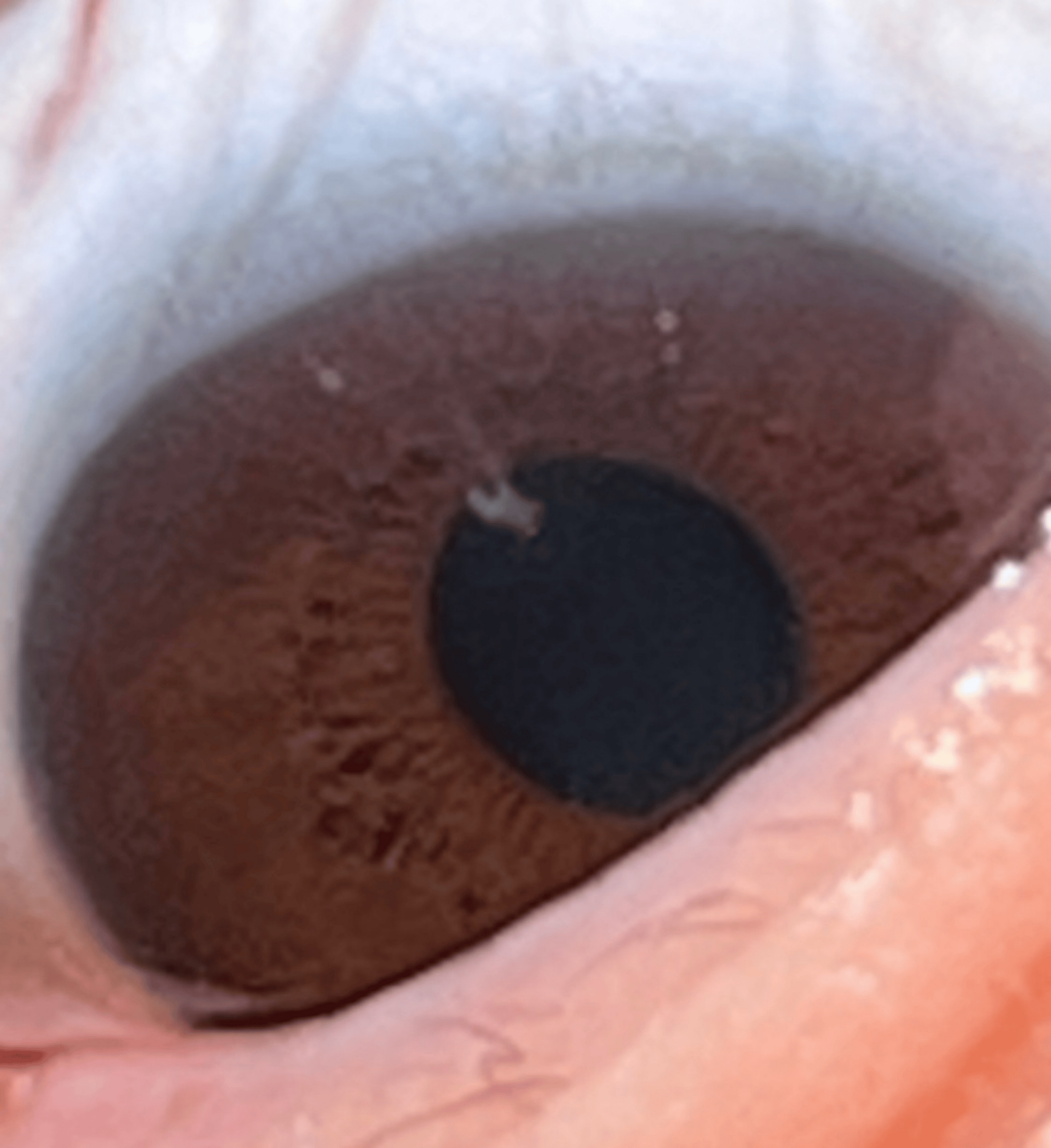
Recurrent corneal erosion of the inferior paracentral cornea, September 2024

In September 2024, he continued to experience ocular surface issues, manifesting as recurrent corneal erosions (Figure 2). Despite these symptoms, he remained on lorazepam 2 mg nightly and continued adjunctive use of imipramine and diphenhydramine for breakthrough insomnia. His symptoms consistently worsened on 2 mg lorazepam nights and improved on 1 mg or off-nights across multiple weeks of home monitoring, aligning with a reproducible dose-dependent symptom intensification pattern. Additionally, nocturnal nasal obstruction persisted and was promptly relieved by a few drops of water or saline, a pattern more consistent with mucosal dryness than inflammatory rhinitis.

At his most recent visit (October 2025), his BP measured 95/70 mmHg seated and 92/67 mmHg standing (values below diagnostic thresholds for classical OH), but he still experienced OI symptoms. Laboratory results continued to show no metabolic, electrolyte, renal, hepatic, or thyroid abnormalities, though mild hyperlipidemia and vitamin D insufficiency were noted (Table 1). On further history, he reported irregular daytime hydration with long intervals between drinks, especially on busy days. Urinalysis (UA) on high-symptom days showed specific gravity 1.027, ketonuria (1+), and hyaline casts (3-5 per low-power field (LPF)) (Table 2). We later interpreted this pattern, in the context of his irregular hydration habits, as consistent with relative dehydration acting as a symptom amplifier. In addition, orthostatic BP and HR measurements showed ΔSBP modestly increasing and ΔHR rising more significantly on 2 mg lorazepam nights, with symptom reproduction; changes were smaller with fewer symptoms on 1 mg or off-nights. The ACB from co-medications was approximately 6 (imipramine 3 + diphenhydramine 3), highlighting the possible contribution of medication-induced dryness to his symptoms (Table 3). Overall, this pattern of modest BP drops with larger HR increases and reproducible symptom provocation was felt to be more consistent with OI than with classical OH.

**Table 1:** Baseline laboratory evaluation on October 10, 2025: no metabolic or endocrine abnormalities identified CBC: complete blood count; CMP: comprehensive metabolic panel; ECG: electrocardiogram; TSH: thyroid-stimulating hormone

Test	Result	Reference range
CBC	No abnormalities detected	Normal range varies by test
CMP	No abnormalities detected	Normal range varies by test
Thyroid function tests	Normal	TSH: 0.4-4.0 mU/L; free T4: 0.8-1.8 ng/dL
Fasting glucose	92 mg/dL	70-100 mg/dL (normal)
Vitamin B12	Normal	200-900 pg/mL
Morning cortisol	Normal	6-23 µg/dL (varies by time of day)
ECG	Normal sinus rhythm	No abnormalities

**Table 2:** Urinalysis with hydration markers on October 10, 2025 LPF: low-power field

Marker	Result	Reference range
Specific gravity	1.027	1.005-1.030 (normal)
Ketonuria	1+ (intermittent)	Negative
Hyaline casts	3-5/LPF	0-2/LPF (normal)
Protein	Negative	Negative (normal)
Blood	Negative	Negative
pH	5.5	4.5-8.0 (normal)
Leukocyte esterase	Negative	Negative
Nitrite	Negative	Negative
Creatinine	1.2 mg/dL	0.6-1.2 mg/dL (normal)

**Table 3:** Orthostatic vitals and symptom score by lorazepam dose ΔSBP: change in systolic blood pressure between supine and three-minute standing; ΔHR: change in heart rate between supine and three-minute standing; ACB: anticholinergic burden (sum of standardized drug-specific scores); TCA: tricyclic antidepressant; AH: antihistamine Symptom score: 0=no symptoms; 1=mild; 2=moderate; 3=severe

Night	Lorazepam dose (mg)	Supine BP (5 min)	Supine HR (5 min)	Standing 1 min BP	Standing 1 min HR	Standing 3 min BP	Standing 3 min HR	ΔSBP (3 min)	ΔHR (3 min)	Symptom score (0–3)	ACB (imipramine + diphenhydramine)
A	1	95/58	64	93/58	69	92/59	70	-3	6	1	3 (imipramine 1 + diphenhydramine 2)
B	2	96/59	63	89/56	82	87/57	86	-9	18	3	6 (imipramine 3 + diphenhydramine 3)
C	1 (off TCA/AH)	94/55	62	90/58	71	89/59	74	-4	9	1	0 (no TCA/AH)
D	2 (with TCA/AH)	93/56	60	88/55	80	85/56	83	-8	20	3	6 (imipramine 3 + diphenhydramine 3)

## Discussion

BZDs can suppress central sympathetic outflow and blunt baroreflex sensitivity through GABAergic mechanisms, impairing rapid vasoconstrictor responses to standing [7]. Controlled studies in humans show measurable, and in some cases dose-dependent, decrements in autonomic cardiovascular control during BZD exposure, supporting a causal link between agents such as lorazepam and postural BP instability [8]. Population data indicate that older adults using BZDs experience greater immediate systolic BP declines on standing and higher fall risk than non-users, aligning with a model of compromised hemodynamic compensation [9]. In this patient with constitutional low BP, symptom onset soon after BZD initiation, reproducible worsening at 2 mg doses with improvement on 1 mg or off-nights, and persistence despite the absence of alternative laboratory or cardiac explanations provide pharmacologic plausibility for BZD-related OI on a low BP background [10]. Although we did not perform advanced autonomic testing, such as head-up tilt with continuous BP/HR monitoring, transcranial Doppler (TCD), or near-infrared spectroscopy (NIRS), the reproducible temporal association and absence of alternative laboratory or cardiac explanations strengthen causal inference in a real-world setting [11].

Concomitant TCA and first-generation AH use likely added a clinically meaningful ACB, reducing parasympathetic control of nasal and ocular glands and precipitating dryness [12]. Anticholinergic-induced nasal mucosal dehydration can present as paradoxical nocturnal obstruction that rapidly improves with hydration, a pattern distinct from allergic rhinitis [13]. Chronic systemic anticholinergic exposure reduces tear-film secretion, promotes tear-film instability, and delays corneal epithelial healing, providing a plausible mechanistic pathway for recurrent erosions after corneal refractive surgery in susceptible patients [14]. Taken together, BZD-mediated autonomic suppression and anticholinergic mucosal effects constitute a physiologic double hit that destabilizes both cardiovascular and secretory homeostasis in low BP phenotypes [15]. Clinically, clinicians should evaluate baseline autonomic vulnerability (resting BP, family history) before initiating sedatives, avoid simultaneous BZD-TCA-AH combinations when possible, prioritize cognitive behavioral therapy for insomnia (CBT-I) and other non-pharmacologic approaches, and follow longitudinal orthostatic vitals with patient education on posture transitions.

Framing and mechanisms

In this case, OI on a low BP background best explains symptom reproduction with HR increases despite modest BP deltas. A low resting BP suggests limited baroreflex reserve, making small postural hemodynamic shifts symptomatic when GABAergic dampening is present [16].

Causality signals

Beyond temporality, the patient's logs captured dose-response (worse on 2 mg), dechallenge/re-challenge (improvement on 1 mg/off-nights with recurrence on re-exposure), and partial de-confounding (association persisting on days without TCA/AH). These features strengthen real-world inference [17].

Hydration/modulators

UA concentration with intermittent ketonuria and 3-5 hyaline casts/LPF, in the setting of irregular daytime hydration with long intervals between drinks, indicates relative dehydration as a trigger that amplified OI on susceptible days. Counseling emphasized structured hydration, modest salt liberalization as tolerated, gradual posture transitions, and optional compression garments [18].

Headache phenotype

The evening headaches occurring after orthostatic symptoms, with transient visual blurring, fit a low-flow/orthostatic hypoperfusion mechanism over primary tension-type. Unlike spontaneous intracranial hypotension and other postural headache syndromes, there were no imaging features or clinical signs of cerebrospinal fluid (CSF) leak, and symptoms tracked more closely with lorazepam dosing and OI than with sustained positional CSF pressure changes. Medication-overuse headache was unlikely by pattern but remains on routine review [19].

Management implications

Practical safeguards for low BP phenotypes include minimizing or avoiding 2 mg nights, aligning dosing with orthostatic BP/HR logs, reducing total ACB (ACB ≈ 6 on co-med nights), and CBT-I-first care. When medication is necessary, options are weighed for orthostatic/anticholinergic risk (e.g., very-low-dose doxepin with orthostatic caution; trazodone with reported orthostatic hypotension; mirtazapine with sedation/rare orthostatic hypotension; orexin antagonists with generally modest hemodynamic impact but potential daytime somnolence) [20].

## Conclusions

This case highlights a pragmatic triad of pharmacologic autonomic suppression in a patient with baseline hypotension: BZD-associated OI on a low BP background, secondary headache likely from transient cerebral hypoperfusion, and a plausible contribution of anticholinergic-associated mucosal/ocular dryness. These observations are particularly relevant for patients with resting low BP or familial hypotension who are exposed to multiple sedatives and anticholinergic agents, in whom sedative polypharmacy can quietly erode homeostatic control. Careful dose regulation, avoidance of redundant sedatives, preference for behavioral sleep therapies, and routine orthostatic monitoring are key to prevention and safer long-term insomnia management.
